# Effect of Different Knee Braces in ACL-Deficient Patients

**DOI:** 10.3389/fbioe.2020.00964

**Published:** 2020-08-26

**Authors:** Anne Focke, Hannah Steingrebe, Felix Möhler, Steffen Ringhof, Stefan Sell, Wolfgang Potthast, Thorsten Stein

**Affiliations:** ^1^BioMotion Center, Institute of Sports and Sports Science, Karlsruhe Institute of Technology (KIT), Karlsruhe, Germany; ^2^Sports Orthopedics, Institute of Sports and Sports Science, Karlsruhe Institute of Technology (KIT), Karlsruhe, Germany; ^3^Department of Sport and Sport Science, University of Freiburg, Freiburg, Germany; ^4^Joint Center Black Forest, Neuenbürg, Germany; ^5^Institute of Biomechanics and Orthopaedics, German Sport University Cologne, Cologne, Germany; ^6^ARCUS Clinics Pforzheim, Pforzheim, Germany

**Keywords:** rigid, soft, walking, cutting, kinematics, 3D, knee joint

## Abstract

Knee braces are often used during rehabilitation after ACL injury. There are two main concepts, rigid and soft braces, but studies comparing the two show conflicting results. Most studies used movement tasks with low translational or rotational loads and did not provide joint kinematics. Therefore, the purpose of this study was to investigate the influence of two different knee braces (rigid vs. soft) on knee joint kinematics in ACL-deficient patients compared to an unbraced control condition using two tasks (walking and 180° cutting) provoking knee movements in the frontal and transverse planes. 17 subjects with ACL-deficient knees participated in this study. 3D knee joint kinematics were recorded. To provoke frontal plane knee joint motion a laterally tilting plate was applied during a walking task. Both braces reduced the maximum valgus angle compared to the unbraced condition, stabilizing the knee joint against excessive valgus motion. Yet, no differences in peak abduction angle between the two braces were found. However, a significant extension deficit was observed with the rigid brace. Moreover, both braces increased transverse plane RoM and peak internal rotation angle, with the effects being significantly larger with the rigid brace. These effects have been associated with decreased knee stability and unphysiological cartilage loading. Therefore, the soft brace seems to be able to limit peak abduction with a lesser impact on physiological gait compared to the rigid brace. The cutting task was selected to provoke transverse plane knee movement and large external knee rotation was expected. However, none of the braces was able to reduce peak external knee rotation. Again, an increase in transverse plane RoM was observed with both braces. Based on these results, no brace outmatched the other in the second task. This study was the first attempt to clarify the effect of brace design for the stabilization of the knee joint during movements with frontal and transverse plane loading. However, to provide physicians and patients with a comprehensive guideline for brace usage, future studies will have to extent these findings to other daily or sportive movement tasks.

## Introduction

The knee joint is of great importance for human locomotion and is one of the most complex joints of the entire body (Tittel, 2003). Its anatomy does not provide bony guidance for rotation and extreme translation between the tibia and femur; therefore the ligamentous apparatus of the knee is not only very important, but also vulnerable in these situations (Tittel, 2003). Ruptures of the anterior cruciate ligament (ACL) are the most common ligament injury in the knee (Palm et al., 2012). Approximately 200,000 ACL injuries occur each year in the United States (Woo et al., 2006b; Escamilla et al., 2012; Strutzenberger et al., 2012). Most of these injuries are caused by non-contact situations (Spindler and Wright, 2008; Levine et al., 2013), and females show a higher injury rate than males (Woo et al., 2006a; Spindler and Wright, 2008). The highest injury rates are seen in sports which include stop-and-go actions, jumps, rotations and fast changes of velocity or direction such as football, handball, basketball, volleyball, skiing and tennis (Lam et al., 2009; Levine et al., 2013). ACL ruptures typically occur during movements with high knee valgus moments in combination with internal or external rotations of the tibia (Hughes and Watkins, 2006).

Consequences of ACL ruptures are biomechanical and neuromuscular changes with impact on the kinematics of the knee joint. Previous studies showed a higher anterior shift of the tibia in the ACL-deficient knee (Beynnon et al., 2003) as well as a higher variability of knee kinematic patterns (Dennis et al., 2005). Besides these mechanical effects, the proprioceptive capacity of the knee joint is also reduced. Due to a decrease in afferent input, reaction times to external disturbances, and thereby postural control mechanisms, are negatively affected (Lysholm et al., 1998; Lee et al., 2009; Palm et al., 2012). Altogether, these changes in biomechanics and the sensorimotor system can lead to compensation mechanisms which result in altered patterns of muscular activity (Théoret and Lamontagne, 2006; Roberts et al., 2007), elevated risk of secondary injuries (Wiggins et al., 2016) and chronic diseases of the overloaded structures (e.g., osteoarthritis) (Andriacchi et al., 2004).

Irrespective of the treatment method (surgical or conservative therapy), braces as a simple and cost-effective aid are often used in order to immobilize the knee joint, to prevent excessive joint movements and to improve stability during activity and thus to prevent secondary injuries. There are several different brace concepts. Traditional knee braces are designed as rigid shells with a hinge joint and straps to mechanically guide and support the knee joint during motion. Previous studies investigating the mechanical effects of such braces showed conflicting results. On the one hand, a reduction of anteroposterior laxity in the knee was observed for low-load conditions (Wojtys et al., 1996; Beynnon et al., 2003). On the other hand, no positive effects of braces on knee stability could be found in more complex conditions or in sports with higher loads (Ramsey et al., 2001; Beynnon et al., 2003). Additionally, functional knee bracing with rigid braces seemed to impact the gait pattern (DeVita et al., 1998). Finally, the subjective perception of comfort differed among patients: while some patients reported discomfort using rigid braces (Risberg et al., 1999; Singer and Lamontagne, 2008), other patients reported benefits such as a higher sense of stability or increased performance (Swirtun et al., 2005; Birmingham et al., 2008).

Due to the conflicting results regarding the effectiveness of rigid braces, alternative brace concepts are brought into focus. Besides pure mechanical stabilization, recent approaches included sensorimotor aspects to potentially enhance stabilization during dynamic situations. This approach was based on previous studies showing that bandages improved sensorimotor control by increasing the proprioception of the muscles surrounding the knee (Beynnon et al., 1999, 2002; Selfe et al., 2008, 2011; Baltaci et al., 2011; Bodendorfer et al., 2019). For patients with ACL ruptures, the disadvantage of bandages seems to be an insufficient mechanical stabilization compared to rigid braces (Luber et al., 1998). Therefore, an alternative to both bandages and rigid braces might be soft braces: these comprise stretchable stocking fabric (similar to bandages) with additional lateral rigid rails (Giotis et al., 2013; Pierrat et al., 2015). Soft braces, comprising bandage fabrics and rigid elements, might therefore combine the benefits of a mechanically stable rigid brace with the proprioceptive advantages of a bandage.

Yet, previous studies comparing rigid and soft braces for the treatment of ACL-deficient subjects show conflicting results. Strutzenberger et al. (2012) found a higher rate of force development in counter-movement jumps and a reduced sway path length during single leg stance on an unstable, laterally perturbed platform with a soft compared to a rigid brace. Beynnon et al. (2003) compared two rigid braces and one soft brace and found a significant reduction in anteroposterior laxity during tests with the Vermont Knee Laxity Device for all three braces. However, positive effects were only found during weight-bearing and non-weight-bearing postures and not for the load acceptance phase. Mortaza et al. (2013) compared a rigid brace, a soft brace and a bandage and found no significant differences in jump distance, peak torque and power between the three conditions during functional (cross-over hop and single leg vertical jump) and isokinetic tests.

The abovementioned studies used movement tasks such as jump, balance or strength tests with low translational and/or rotational loads. Therefore, they do not strain the knee joint in the frontal and transverse planes. These motions, however, are of particular relevance as the function of the ACL is to restrict excessive motion in these planes. Additionally, excessive valgus moments and external and internal rotations of the tibia are known to be the main causes of ACL injuries (Hughes and Watkins, 2006). Consequently, knowledge of the ability of different brace concepts to provide stability of the knee joint during dynamic situations with high frontal and rotational loads is of great interest. The aforementioned studies quantified brace effects mostly using the performance in functional or strength tests and did not provide joint kinematics of the lower extremities. Yet, kinematic data is needed to understand the mode of action of different brace concepts, how they affect gait patterns and to evaluate whether one brace concept provides better knee stabilization effects.

Therefore, the purpose of this study was to investigate the influence of two different braces (rigid vs. soft) on knee joint kinematics in ACL-deficient patients using two movement tasks provoking knee movements in the frontal and transverse planes. It was hypothesized that both braces would stabilize the knee joint, in terms of decreased peak abduction and rotation angles, compared to an unbraced control condition.

## Materials and Methods

### Subjects

During subject recruitment 118 potential subjects were screened for eligibility. Thereof 41 fulfilled all defined inclusion criteria and were invited for the first test session. During this session a total of 17 subjects with ACL-deficient knees demonstrated an unstable knee joint and subsequently participated in this study (10 females, 7 males; age: 44.4 ± 11.5 years; height: 1.68 ± 0.08 m; mass: 77.6 ± 11.5 kg). Subjects were sportively active for 198 ± 117 min per week with focus on either team sports (e.g., handball, football) or recreational sportive activities (e.g., running, cycling, hiking, or swimming). The time interval between injury and biomechanical data collection was between 0.25 and 32 years (11.8 ± 12.6 years). Although some of the ACL ruptures had occurred several years ago, all subjects showed symptoms of an unstable knee. Knee instability was defined as fulfilling at least two of the following three criteria: (a) side-to-side difference in knee laxity ≥ 3 mm evaluated by use of the KT-1000TM arthrometer (MEDmetric, San Diego, CA, United States), (b) limb symmetry score below 85% during both the single hop test for distance, and (c) the timed hop test (Noyes et al., 1991). Besides knee instability, additional inclusion criteria were: (a) unilateral rupture of the ACL without surgical reconstruction, (b) age between 18 and 60 years, (c) moderate sport activity, (d) absence of injuries of the posterior cruciate ligament or other structures in the knee, (e) no gonarthrosis of grade 2–4 (Kellgren and Lawrence, 1957), and (f) contralateral side free of injuries.

The study design was approved by the Ethics Board of the State Medical Association of Baden-Württemberg. All patients were informed about the procedures of the study and gave their written informed consent prior to study participation.

### Experimental Protocol

All subjects were tested on two occasions. During the first session, patients were informed about the study procedure and were screened regarding the inclusion criteria via questionnaires. Then, knee instability was tested using the abovementioned tests. Subjects included in the study were provided with both a soft brace (SofTec Genu; Bauerfeind Inc., Zeulenroda-Triebes, Germany) and a rigid brace (4Titude Donjoy; ORMED GmbH, Freiburg, Germany). Both braces were fitted individually to the injured knee by an experienced orthopedic technician, and subjects were instructed regarding the correct positioning of the braces. Subsequently, patients were familiarized with the movement tasks to reduce learning effects. Subjects then wore both braces in alternation during their everyday activities for a period of at least four weeks to avoid habituation effects during measurement.

During the second session, subjects completed a standardized 5 min warm-up on a bicycle ergometer (intensity: 50% body mass in Watt, 60 RPM). They then performed two movement tasks: (a) walking over a suddenly tilting plate and (b) 180° cutting. These tasks were chosen to provoke external moments in the frontal and transverse planes, respectively, and therefore to induce instability and relative motion between femur and tibia. Each patient performed both movement tasks under three different conditions: injured leg with rigid brace, injured leg with soft brace, and injured leg without brace. The order of the three conditions within each movement task was randomized for all subjects.

**FIGURE 1:**
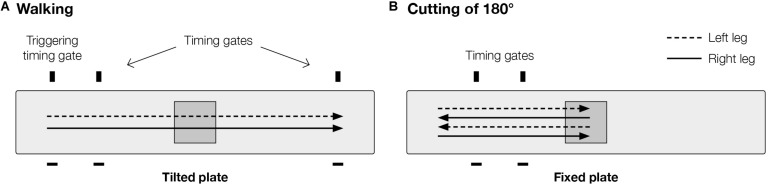
Experimental setup of movement tasks: **(A)** walking, and **(B)** 180° cutting. Plate contact occurred always with injured leg. Cutting movement **(B)** was performed in a step turn manner.

Walking over tiltable plate: The subjects were instructed to walk across a 7.5 m walkway at a prescribed speed of 5 km/h (± 5%), verified using infrared timing gates (Figure 1A). A custom-made tiltable plate (60 × 60 cm) was embedded in the middle of the walkway, which had to be struck by the subjects with their injured leg. Hydraulic controlled tilting of the plate by 9° either to the left or to the right side, provoking a supination or pronation at the ankle, was triggered by another infrared timing gate positioned at the beginning of the walkway. The time delay between triggering and onset of tilting was set by a custom-made software program allowing for two different conditions: the plate was either already completely tilted before being struck (predictive condition) or the plate tilted when it was struck (reactive condition). Both time delay (predictive or reactive) and tilting direction (pronation or supination) were randomized within each of the three brace conditions. Hence, subjects were unaware of the tilting condition prior to movement initiation. For each test condition, three valid trials were recorded. Thus, a total of 36 successful trials were recorded: 2 time delay × 2 tilting directions × 3 brace conditions × 3 trials. However, since valgus movements are more important than varus movements in the context of ACL injuries, only pronation trials (18 trials) were analyzed in this paper.

Cutting of 180°: Subjects performed a cutting movement of 180° after walking with a prescribed approach speed of 7 km/h (± 5%). The experimental setup (Figure 1B) was very similar to the walking task, however, the plate embedded in the middle of the walkway was fixed in a straight position rather than tilting. The distance from initial position to the plate was 3.5 m. Two timing gates were positioned 1 and 2 m in front of the plate and were used to control approach speed. The cutting movement was performed on the plate in a step turn manner using the injured leg. Three valid trials were recorded for each test condition resulting in a total of 9 recorded trials (3 brace conditions × 3 trials).

### Data Collection

**FIGURE 2:**
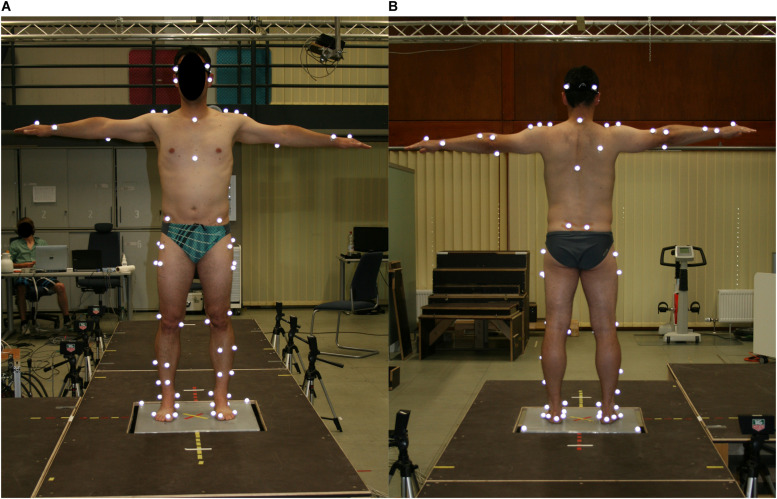
Marker positions in frontal **(A)** and dorsal **(B)** view.

A motion capture system (10 cameras; 200 Hz; Vicon Motion Systems; Oxford Metrics Group, Oxford, United Kingdom) was used to capture 42 spherical retro-reflective markers (14 mm) placed on predefined anatomical landmarks of the subject (Figures 2A,B). In addition 22 anthropometric measurements were taken manually according to the alaska Dynamicus Handbook [Advanced Lagrangian Solver in kinetic Analysis, Insys GmbH, Chemnitz, Germany (Härtel and Hermsdorf, 2006)]. A static trial was recorded during which the participants stood in a neutral position, with their feet shoulder-width apart, toes pointing anteriorly and hip and knee joints in full extension. This static trial was used to adapt the multi-body-model (alaska Dynamicus) to each subject. Three dimensional ground reaction forces were captured with the custom-made tiltable plate embedded in the middle of the walkway.

### Knee Marker Reconstruction

When the braces were applied, the knee markers at the injured leg were removed during the dynamic trials. Therefore, additional clusters of four markers were attached to the thigh and shank to reconstruct the knee markers. During the initial static reference trial, the actual knee markers were applied in addition to the cluster markers. The cluster marker positions defined a reference frame, which was embedded rigidly to the shank (shank reference frame, SF). This frame was also used to set up reference vectors from the SF origin to the respective knee marker $\tilde{b}$ and to each cluster marker ${\tilde{x}}_{i}$ (i = 1,…,4). Both $\tilde{b}$ and ${\tilde{x}}_{i}$ can be assumed to be time-invariant in SF.

Once the reference vectors were determined by a reference trial, knee markers were removed. For the trials without the knee markers a least-squares algorithm (Cappozzo et al., 1997) was used to calculate the optimized position and orientation of SF. At first, SF was set up identically to the reference trial at each time instant. Optimization was achieved by minimizing the deviation *e*_*i*_ between each instantaneous cluster marker position *x*_*i*_ in the current SF in relation to the corresponding reference vector ${\tilde{x}}_{i}$:

$$e_{i}={\tilde{x}}_{i}-x_{i}.$$

To transform this equation from SF to the laboratory frame (LF), a translation vector *p* and a rotation matrix *R* were introduced, so that

$$e_{i}=R\,{\tilde{x}}_{i}-\left(y_{i}-p\right)$$

with *y*_*i*_ as the coordinates of the cluster markers in the LF and *p* as the vector pointing from the origin of the LF to the origin of the SF. Solving $\min_{p,R}\sum_{\text{i}=1}^{4}{||e_{i}||}^{2}$ allowed calculation of *p* and *R* by a singular value decomposition (Hanson and Norris, 1981). Together *p* and *R* transformed SF to an optimized SF.’ The required knee marker position *c* in the LF could be calculated at each time instant by a retransformation of the reference vector $\tilde{b}$ (time-invariant in SF’) to LF:

$$c=R\,\tilde{b}+p.$$

This procedure was done for both medial and lateral knee markers for all three brace conditions.

### Data Processing and Analysis

Kinematic data were analyzed during stance phase on the plate. Heel-strike and toe-off of the injured leg on the plate were determined via force sensors embedded in the plate using a threshold of 10 N (Tirosh and Sparrow, 2003). Force data were filtered with a third-order Butterworth low-pass filter with a cut-off frequency of 50 Hz. Three-dimensional marker trajectories were filtered using a second-order Butterworth low-pass filter with a cut-off frequency of 6 Hz for the walking condition and 10 Hz for the cutting condition (Kirtley, 2006). An inverse kinematics approach using the multi-body model Dynamicus (Härtel and Hermsdorf, 2006) was used to calculate 3D knee angles as objective parameters suggested in the literature to be indicators for knee joint stability (Schrijvers et al., 2019). Based on the preprocessed data, peak joint angles (minimum and maximum), ranges of motion (RoM), joint angles at touch down (TD) and at resultant peak ground reaction force (Peak GRF) in the sagittal, frontal and transverse planes were calculated for the knee joint during the stance phases of walking and cutting. The RoM was used to eliminate possible errors of absolute measures (Giotis et al., 2013), TD was used to indicate phase of load transfer and Peak GRF was taken as the time of peak load, as previously used (Ewing et al., 2016; Alirezaei Noghondar and Bressel, 2017; Duffell et al., 2017). For all parameters the main focus was on the frontal and transverse plane as ACL-deficient knees are particularly vulnerable to forces in these planes (Hughes and Watkins, 2006; Levine et al., 2013). The values acquired from the three valid trials were averaged for each test condition.

### Statistics

All statistical tests were performed using IBM SPSS Statistics 24.0 (IBM Corporation, Armonk, NY, United States). The effect of braces (rigid, soft, without) on knee angles was investigated by use of one-way repeated measures ANOVA tests, separately run for the walking conditions (predictive, reactive) and the 180° cutting movement. If sphericity was violated, Greenhouse-Geisser estimates were used to correct for these violations. The significant main effects for the braced conditions were analyzed in post-hoc comparisons with Holm-Bonferroni corrections to adjust for multiple comparisons. Effect sizes were determined using partial eta squared (small effect: $\eta_{p}^{2}$ = 0.01; medium effect: $\eta_{p}^{2}$ = 0.06; large effect $\eta_{p}^{2}$ = 0.14) (Cohen, 1988; Richardson, 2011). For all statistical tests, the level of significance was set *a priori* to 0.05.

## Results

**FIGURE 3:**
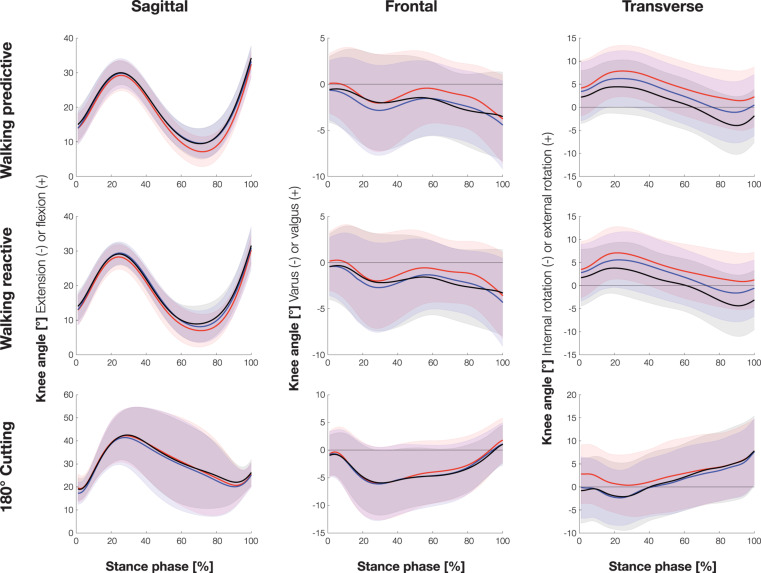
Knee joint kinematics of all three movement tasks in the sagittal, frontal, and transverse planes during stance phase (mean ± sd). Red = without brace, black = rigid brace, blue = soft brace.

Figure 3 shows the knee joint kinematics during predictive and reactive walking as well as 180° cutting in the sagittal, frontal and transverse planes during stance phase. Tables 1–3 present the mean values of knee joint angles during stance phase of all three movement tasks. Data are given in all planes for all brace conditions with respective *p*-values and effect sizes.

**TABLE 1:** Mean values and standard deviations of knee joint angles [°] during stance phase of predictive walking (force plate tilted before the step) with respective *p*-values and effect sizes as revealed by one-way repeated measures ANOVAs and Holm-Bonferroni corrected pairwise comparisons.

	Rigid	Soft	Without	ANOVA	Rigid vs. soft	Rigid vs. without	Soft vs. without
Variable	Mean (sd)	Mean (sd)	Mean (sd)	*p* ($\eta_{p}^{2}$)	*p*	*p*	*p*
**Sagittal**							
RoM	27.34 (3.86)	27.16 (4.38)	28.18 (3.67)	0.084 (0.144)			
Min.	9.04 (4.10)	8.85 (4.06)	6.87 (4.06)	<0.001(0.468)*	0.636	0.002*	<0.001*
Max.	36.38 (3.02)	36.01 (3.38)	35.05 (3.13)	0.044(0.178)*	0.527	0.045*	0.148
At TD	15.01 (4.52)	13.96 (4.60)	13.92 (4.10)	0.048(0.193)*	0.160	0.048*	0.924
At Peak GRF	29.77 (3.37)	29.58 (4.17)	28.97 (4.41)	0.255 (0.082)			
**Frontal**							
RoM	4.47 (2.07)	5.03 (1.92)	5.06 (1.89)	0.083 (0.162)			
Min.	−4.40(4.52)	−5.08(4.59)	−4.24(4.70)	0.006(0.271)*	0.044*	0.548	0.009*
Max.	0.07 (4.19)	−0.05(3.66)	0.83 (3.73)	0.005(0.278)*	0.699	0.016*	0.006*
At TD	−0.57(3.49)	−0.66(3.13)	0.09 (3.16)	0.034(0.191)*	0.818	0.039*	0.039*
At Peak GRF	−1.75(5.06)	−2.62(4.73)	−1.76(5.04)	0.003(0.301)*	0.030*	0.971	0.030*
**Transverse**							
RoM	9.42 (2.52)	8.43 (2.50)	7.56 (2.24)	<0.001(0.487)*	0.014*	<0.001*	0.019*
Min.	−4.33(6.03)	−1.72(6.34)	0.69 (6.35)	<0.001(0.631)*	0.008*	<0.001*	0.008*
Max.	5.09 (5.48)	6.72 (5.60)	8.26 (5.35)	<0.001(0.400)*	0.061	<0.001*	0.040*
At TD	2.20 (5.57)	3.42 (6.16)	4.17 (5.82)	0.027(0.202)*	0.258	0.033*	0.262
At Peak GRF	4.45 (5.67)	6.23 (5.85)	7.85 (5.39)	<0.001(0.413)*	0.056	<0.001*	0.056

*sd = standard deviation; RoM = range of motion; TD = touch down; GRF = ground reaction force; sagittal: (+) = flexion, (−) = extension; frontal: (+) = valgus, (−) = varus; transversal: (+) = external rotation, (−) = internal rotation; level of significance = 0.05.*

### Walking

As tilting of the plate was intended to induce perturbations in the frontal plane, knee valgus and varus angles (particularly maximum valgus angles) were considered the main outcome parameters for walking.

Maximum valgus angle mainly occurred at the beginning of stance phase and was therefore similar to the valgus angle at TD. Both braces significantly reduced the maximum valgus angle compared to the unbraced condition for both walking conditions (predictive and reactive) (Tables 1, 2). The valgus angle at TD was also significantly smaller with both braces for predictive walking, and a comparable tendency was found for reactive walking (Tables 1, 2).

**TABLE 2:** Mean values and standard deviations of knee joint angles [°] during stance phase of reactive walking (force plate tilted during the step) with respective *p*-values and effect sizes as revealed by one-way repeated measures ANOVAs and Holm-Bonferroni corrected pairwise comparisons.

	Rigid	Soft	Without	ANOVA	Rigid vs. soft	Rigid vs. without	Soft vs. without
Variable	Mean (sd)	Mean (sd)	Mean (sd)	*p* ($\eta_{p}^{2}$)	*p*	*p*	*p*
**Sagittal**							
RoM	26.52 (5.72)	27.43 (4.47)	27.15 (4.00)	0.533 (0.039)			
Min.	8.22 (4.97)	7.34 (4.19)	6.36 (4.55)	0.002(0.334)*	0.089	0.012*	0.018*
Max.	34.74 (3.73)	34.78 (3.61)	33.51 (3.63)	0.202 (0.095)			
At TD	14.01 (4.14)	13.04 (4.24)	12.91 (4.14)	0.036(0.187)*	0.156	0.042*	0.765
At Peak GRF	28.88 (3.21)	29.12 (3.11)	28.06 (3.58)	0.064 (0.158)			
**Frontal**							
RoM	4.36 (1.59)	5.19 (1.90)	4.86 (1.69)	0.018(0.221)*	0.051	0.182	0.184
Min.	−4.21(4.40)	−5.11(4.56)	−4.07(4.58)	0.007(0.264)*	0.044*	0.643	0.030*
Max.	0.15 (4.10)	0.09 (3.51)	0.79 (3.66)	0.024(0.208)*	0.837	0.030*	0.030*
At TD	−0.45(3.47)	−0.43(3.07)	0.16 (3.11)	0.059 (0.162)			
At Peak GRF	−1.88(5.08)	−2.48(4.67)	−1.73(4.85)	0.024(0.208)*	0.126	0.490	0.063
**Transverse**							
RoM	9.10 (2.52)	8.16 (2.42)	7.46 (2.20)	<0.001(0.444)*	0.028*	<0.001*	0.034*
Min.	−4.78(6.41)	−2.07(6.17)	0.03 (6.12)	<0.001(0.614)*	0.006*	<0.001*	0.006*
Max.	4.32 (5.42)	6.08 (5.74)	7.49 (5.46)	0.001(0.364)*	0.064	0.003*	0.064
At TD	1.72 (6.00)	2.81 (5.97)	3.50 (5.93)	0.055 (0.166)			
At Peak GRF	3.67 (5.54)	5.54 (5.85)	7.10 (5.53)	<0.001(0.381)*	0.068	<0.001*	0.068

*sd = standard deviation; RoM = range of motion; TD = touch down; GRF = ground reaction force; sagittal: (+) = flexion, (−) = extension; frontal: (+) = valgus, (−) = varus; transversal: (+) = external rotation, (−) = internal rotation; level of significance = 0.05.*

Significantly different results between the braced and unbraced conditions were also found in the sagittal and transverse planes. Patients generally walked with a more flexed knee in the braced conditions compared to the unbraced condition, whereby mainly the rigid brace showed significant and larger differences to the unbraced condition than the soft brace (Tables 1, 2).

In the transverse plane, the maximum external rotation angle and the rotation angle at Peak GRF occurred at similar times and were significantly smaller for the rigid than the unbraced condition in predictive and reactive walking (Tables 1, 2). The soft brace significantly reduced only the maximum external rotation angle in predictive walking compared to the unbraced condition (Tables 1, 2). The rotation angle at TD was significantly smaller for the rigid brace than the unbraced condition in predictive but not reactive walking (Tables 1, 2). With both braces a significant increase in transverse plane RoM and peak internal rotation occurred compared to the unbraced condition. These alterations were significantly larger with the rigid brace compared to the soft brace.

### Cutting of 180°

During cutting, stabilization of the knee joint in the transverse plane is of particular relevance. Specifically, at the beginning of the stance phase significant differences between braced and unbraced conditions were found in the transverse plane. External rotation angle at TD and Peak GRF were significantly smaller for both braces than the unbraced condition (Table 3). Maximum external rotation angle occurred at the end of stance phase and was similar for all three conditions. Again, both braces increased the observed RoM and additionally, a significant increase in peak internal rotation occurred with the rigid brace compared to the control condition.

**TABLE 3:** Mean values and standard deviations of knee joint angles [°] during stance phase of cutting with respective *p*-values and effect sizes as revealed by one-way repeated measures ANOVAs and Holm-Bonferroni corrected pairwise comparisons.

	Rigid	Soft	Without	ANOVA	Rigid vs. soft	Rigid vs. without	Soft vs. without
Variable	Mean (sd)	Mean (sd)	Mean (sd)	*p* ($\eta_{p}^{2}$)	*p*	*p*	*p*
**Sagittal**							
RoM	28.92 (10.34)	30.07 (10.80)	30.51 (10.35)	0.291 (0.074)			
Min.	15.91 (5.76)	13.98 (5.97)	14.68 (6.40)	0.014(0.233)*	0.048*	0.048*	0.306
Max.	44.83 (12.22)	44.05 (13.16)	45.19 (12.63)	0.597 (0.032)			
At TD	19.13 (4.82)	17.33 (4.70)	19.59 (5.88)	0.004(0.292)*	0.028*	0.440	0.021*
At Peak GRF	35.45 (8.77)	35.24 (9.10)	35.96 (7.67)	0.862 (0.009)			
**Frontal**							
RoM	8.12 (3.28)	8.56 (3.45)	9.18 (3.69)	0.004(0.288)*	0.156	0.015*	0.068
Min.	−6.67(5.26)	−7.01(5.94)	−7.01(6.08)	0.474 (0.046)			
Max.	1.45 (3.21)	1.55 (3.64)	2.17 (3.79)	0.018(0.222)*	0.730	0.018*	0.064
At TD	−0.95(3.56)	−0.77(4.00)	−0.71(3.99)	0.689 (0.023)			
At Peak GRF	−3.86(5.00)	−4.11(5.76)	−4.20(5.62)	0.538 (0.038)			
**Transverse**							
RoM	12.38 (4.48)	12.62 (4.01)	10.43 (3.22)	0.001(0.338)*	0.675	0.026*	<0.001*
Min.	−3.69(7.12)	−4.08(6.04)	−1.71(6.57)	0.023(0.210)*	0.693	0.009*	0.058
Max.	8.70 (7.26)	8.54 (6.41)	8.72 (6.71)	0.979 (0.001)			
At TD	−0.80(6.84)	−0.10(6.47)	2.80 (6.19)	<0.001(0.391)*	0.492	<0.001*	0.010*
At Peak GRF	−1.47(7.05)	−1.55(6.09)	1.31 (6.13)	0.011(0.285)*	0.941	<0.001*	0.020*

*sd = standard deviation; RoM = range of motion; TD = touch down; GRF = ground reaction force; sagittal: (+) = flexion, (−) = extension; frontal: (+) = valgus, (−) = varus; transversal: (+) = external rotation, (−) = internal rotation; level of significance = 0.05.*

## Discussion

The present study investigated the effects of two different knee brace designs, soft and rigid, on knee joint kinematics in ACL-deficient patients. In summary, results showed that both braces induced changes in knee joint kinematics during walking and cutting when compared to an unbraced control condition.

With regard to the walking task, in which the tilted plate was used to disturb joint stability in the frontal plane, both braces were able to reduce the maximum valgus angle as compared to the unbraced condition, stabilizing the knee joint against an excessive valgus motion during stance phase. Despite their differing concepts, both rigid and soft brace demonstrated that they provide their desired effects. However, both braces revealed a higher RoM in transverse plane caused by a more pronounced internal rotation compared to the unbraced condition. This effect was more pronounced in the rigid compared to the soft brace.

The cutting task was conducted to provoke relative rotation between the proximal and distal segments of the ACL-deficient knee joint and therefore, to evaluate the compensatory capacities of the braces in the transverse plane. In this regard, none of the braces was able to reduce peak external knee rotation. Yet, again a significant increase in transverse plane RoM was observed with both braces, caused by a more pronounced internal rotation.

### Embedding of the Results Into the Current State of Research

Due to large differences between the current study and previous work in terms of the selected movement tasks, applied methods, calculated parameters, included subjects and braces, a comparison of the results is extremely difficult. Previous studies observed a reduction in anteroposterior knee joint laxity (Wojtys et al., 1996; Beynnon et al., 2003; Strutzenberger et al., 2012; Pierrat et al., 2015) for low-load conditions and lower ranges of motions in the frontal and transverse planes during running (Théoret and Lamontagne, 2006). Furthermore, significant performance benefits in terms of improved dynamic balance (Palm et al., 2012; Strutzenberger et al., 2012) and increased lower limb rate of force development (Strutzenberger et al., 2012) were found for braced compared to unbraced control conditions. Stabilization of the knee joint against excessive translational and rotational motions was mainly shown in studies using rigid braces (Wojtys et al., 1996; Beynnon et al., 2003; Théoret and Lamontagne, 2006; Strutzenberger et al., 2012). These braces are primarily used to mechanically guide the knee joint and thus to prevent secondary injuries resulting from excessive joint loads. Particularly in ACL-deficient knees, the brace is supposed to undertake the task of an intact ACL, compensating for the decreased joint stability and the increased variance in joint kinematics (Beynnon et al., 2003; Dennis et al., 2005). In contrast, improvements in postural control and muscular strength were mainly seen in studies using soft braces. Specifically when wearing sleeve braces, significant increases in dynamic postural stability (Palm et al., 2012; Strutzenberger et al., 2012) and lower limb peak rate of force development (Strutzenberger et al., 2012), but also significantly reduced tibial rotation (Giotis et al., 2013), were observed in ACL-deficient subjects. The authors suggested that these effects might be caused by the flexible stocking fabric stimulating proprioception of the affected leg. However, the lateral splints and straps included in the soft braces also seem to be of great importance in reducing the relative movement between femur and tibia (Giotis et al., 2013). Conclusive evidence is still lacking, however, as other studies have yielded conflicting results (Beynnon et al., 1999, 2003; Ramsey et al., 2001; Swirtun et al., 2005; Mortaza et al., 2013). Especially under dynamic conditions, most of the studies failed to prove either a short- or long-term effect of bracing (Smith and Davies, 2008; Smith et al., 2014). Yet, it seems that the nature of the gains depended on the brace design.

Previous studies that compared rigid with soft braces also found conflicting results. Beynnon et al. (2003) compared the translation of the tibia relative to the femur, measured with the Vermont Knee Laxity Device, while the knee was unweighted, throughout the transition to weight-bearing and during weight-bearing in ACL-deficient patients. They found no significant effects of the braces during the transition from non-weight-bearing to weight-bearing, but significantly lower anteroposterior laxity values for the braced compared to the unbraced knee during non-weight-bearing and weight-bearing conditions. No significant differences were found between the brace concepts. Singer and Lamontagne (2008) included healthy subjects and found that no brace significantly altered joint moment patterns or angular impulse values. Strutzenberger et al. (2012) found a slight advantage for the soft brace compared to the rigid brace for ACL-deficient patients. In detail, the results of balance and jump tests showed similar effects for both braces in terms of joint laxity and decrease of postural sway in a perturbed situation and after a jump with a 90° rotation; however, higher effect sizes and an increased rate of force development during a counter movement jump were found for the soft brace. A meaningful comparison between these previous studies and ours is difficult as Beynnon et al. (2003) did not perform dynamic everyday tasks, Singer and Lamontagne (2008) investigated only healthy subjects and Strutzenberger et al. (2012) recorded no kinematic data. Therefore, these studies can only be used to a limited extent to make statements on the appropriate use of braces in everyday life and sports in ACL-deficient patients.

The current study was the first to contrast the effects of a rigid and a soft brace on knee joint kinematics in ACL-deficient patients during dynamic situations with high frontal and rotational loads. The chosen tasks provoked movements in the frontal and transverse planes and thus challenged the braces in the planes ACL-deficient knees are particularly vulnerable to (Hughes and Watkins, 2006; Levine et al., 2013); in contrast to many previous studies, which applied only static or simple dynamic tests to assess the effects of functional knee braces in ACL-deficient subjects (Wojtys et al., 1996; Beynnon et al., 1999, 2003; Ramsey et al., 2001; Swirtun et al., 2005; Théoret and Lamontagne, 2006; Palm et al., 2012; Strutzenberger et al., 2012; Mortaza et al., 2013; Pierrat et al., 2015). Additionally, we analyzed brace effects in an ACL-deficient subject population, which is important as many previous studies included only healthy (Singer and Lamontagne, 2008; Baltaci et al., 2011; Giotis et al., 2011; Hanzlíková et al., 2016; Bodendorfer et al., 2019) or ACL-reconstructed subjects (DeVita et al., 1998; Risberg et al., 1999; Birmingham et al., 2008; Smith and Davies, 2008; Giotis et al., 2011, 2016; Hanzlíková et al., 2019). As knee joint instability in these populations is prevented by the original or reconstructed ACL, it is difficult to transfer the results on brace stabilization effects to ACL-deficient patients. Finally, we recorded 3D knee joint kinematics as these have been suggested to indicate knee joint stability (Schrijvers et al., 2019).

Considering the abovementioned results, the present study provides some additional evidence for the functional effectiveness of brace designs when applied to ACL-deficient patients. First, it was shown that during walking tasks with frontal disturbance bracing may decrease peak abduction and external rotation in the knee joint. Therefore, braces could provide additional stability when patients return to sports after ACL ruptures. Second, the present study revealed that both rigid and soft braces had similar effects on joint angles in the frontal and transverse planes. However, the rigid brace showed a stronger reduction of the external rotation compared to the soft brace in the walking condition. This might be explained by the different concepts of the braces. The mechanically guiding rigid brace has shells, straps and hinge joints to stabilize the knee joint against excessive joint motions. Due to its rigid thigh and shank cuffs connecting the two lateral joint splints, the external rotation of the tibia could be reduced mechanically and thus to a greater extend compared to the soft brace with only two lateral splints. Nevertheless, there were also significant reductions in external tibia rotation in the soft brace. Thus, the lateral joint splints combined with the stretchable stocking fabric might provide both mechanical stabilization and improved proprioception and neuromuscular control (Beynnon et al., 1999, 2002; Baltaci et al., 2011; Strutzenberger et al., 2012; Giotis et al., 2013). The latter effects might be of particular relevance, as ACL injury affects not just the mechanics of the knee joint, but also the proprioceptive capacity; and as a consequence, postural control and reaction times to external disturbances (Lysholm et al., 1998; Lee et al., 2009; Palm et al., 2012).

Besides the reduction in external rotation an increase in transverse plane RoM caused by a greater internal tibia rotation was observed for both braces compared to an unbraced control condition during both movement tasks (walking task: soft brace <1°; rigid brace ≈ 1–2°; cutting task: soft brace ≈ 2°; rigid brace ≈ 2°). Increased knee RoM of about 3° has been interpreted as an increase in knee instability in subjects with patellofemoral pain syndrome (Richards et al., 2018). Yet, as comparable studies especially during cutting movements are missing future investigations are needed to confirm this effect. Thereby, particular focus should be paid to changes in muscular stabilization with brace application as alterations in muscle activity due to brace application have been emphasized in a review by Brisson and Lamontagne (2014). In addition, during a 3-month follow-up investigation after ACL reconstruction, usage of a rigid brace has shown to significantly increase thigh atrophy compared with patients in an unbraced group (Risberg et al., 1999). However, it should be noted that no soft brace condition was included in this study. Therefore, altered muscle activity patterns and long-term usage of a rigid brace might cause a decrease in muscular joint stabilization which might not be the case with a soft brace, enhancing neuromuscular control. Concerning the internal tibia rotation, previous studies showed that ACL-deficient subjects tend to show greater internal tibia rotation (Georgoulis et al., 2005). An increase of this deviation caused by brace usage might reinforce the effect of a load shift to previously less loaded cartilage areas and thus, could increase the risk for the development of knee OA (Andriacchi et al., 2006; van de Velde et al., 2009). In future studies, attention should be paid to this issue. Thereby, it is important to check whether these findings can be replicated and are also evident in other movement tasks.

Lastly, wearers of rigid braces often report discomfort and changes in gait patterns (DeVita et al., 1998; Risberg et al., 1999; Singer and Lamontagne, 2008), which also becomes apparent in the extension deficiency observed particularly with the rigid brace in the present study.

### Limitations

It is important to note, that our study has some limitations, which will be discussed in the following in order to give a balanced view of our results.

One limitation of the study is that the magnitudes of the differences between the braced and the unbraced conditions were quite small. They ranged between 1° and 3° and might therefore not be clinically relevant. Actually, there is no previous study reporting minimal clinically important differences (MCID) for knee joint kinematics in the transverse and frontal plane for locomotion tasks. Di Stasi and Snyder-Mackler (2012) report a MCID of 3° for the sagittal plane representing about 10% of the RoM. Transferring this percentage to the smaller RoM observed in the frontal and transverse plane, an alteration of 1° would already exceed this threshold. From a clinical perspective, Andriacchi et al. (2006) demonstrated that an increase in tibia internal rotation resulted in cartilage thinning, potentially leading to secondary knee OA. Even though it cannot be inferred from this data, small kinematic changes of 1° can become crucial for joint health due to the repetitive nature of movements like running and walking, which is accompanied by daily thousand-fold cartilage loading during these activities.

The fact that the knee markers were reconstructed using clusters during the dynamic trials certainly leads to greater inaccuracies in the calculation of knee angles. However, placing the knee markers on the braces would not adequately reproduce the underlying relative movement between the tibia and femur. Also, cutting out the braces would not have been possible due to the lateral rigid splints and potential alterations of the braces’ properties. Therefore, calculation using clusters seemed to us to be the most appropriate and accurate method. Additionally, differences in other studies were of similar magnitude (Singer and Lamontagne, 2008; Giotis et al., 2013), which increases the plausibility of our data.

The interpretation of knee angles in the transverse plane can certainly be regarded as critical due to the multi-body modeling applied. However, movements in the transverse plane are significantly involved in the injury mechanism of an ACL rupture (Hughes and Watkins, 2006; Levine et al., 2013) and should therefore not simply be disregarded as in most previous studies. Ramsey et al. (2001) analyzed knee kinematics of ACL-deficient subjects with and without a rigid brace using bone pins and reported changes in frontal and transverse plane knee RoM of similar magnitude as shown in the present study. Even though their study results vary partly between subjects and the methodology allowed only a small sample size, this similarity suggests that the detected effects are consistent across different measurement and modeling methods.

Additionally, time post injury varied strongly between subjects and partially exceeded several years. In order to maintain a sufficiently large sample size we were unable to limit inclusion criteria on this point increasing the risk for confounding changes that might have occurred over time. Nevertheless, we think that the population investigated in this study is representative of the population for which brace usage is a potential treatment method in order to increase knee stability as the perceived knee instability apparently persist even after 32 years post injury (Mihelic et al., 2011).

### Summary of Results, Practical Implications and Future Research

The aim of our study was to analyze, if the two different knee braces are able to stabilize the knee joint, in terms of decreased peak abduction and rotation angles, compared to an unbraced control condition. To provoke frontal plane knee joint motion a laterally tilting plate was applied during walking. The results confirm our hypothesis, that both braces are able to limit the peak abduction angle compared to an unbraced control condition. In addition, our results revealed no differences in peak abduction angle between the two braces. However, a significant extension deficit was observed during walking with the rigid brace. Despite the fact that the movement task was not designed to provoke large knee rotation, both braces led to increased transverse plane RoM caused by an increased peak internal rotation angle, with the effects being significantly larger with the rigid brace. As described previously, these effects have been associated with decreased knee stability and unphysiological cartilage loading. Therefore, for moderate intensity movement tasks with mainly frontal plane knee loading, the soft brace seems to be able to stabilize ACL relevant peak abduction with a lesser impact on physiological gait compared to the rigid brace. The cutting movement of 180° performed in this study was selected to provoke transverse plane knee movement. As it was performed in a step turn manner especially large external knee rotation was expected (Taylor et al., 2005). In contrast to our expectations, none of the braces was able to reduce peak external knee rotation. Yet, again a significant increase in transverse plane RoM was observed with both braces caused by a more pronounced internal rotation. Based on these results, no brace outmatched the other in the second task.

Future studies will have to address the replication of the observed kinematic changes as well as their extension to other movement tasks like running or stair climbing. Thereby, special attention should be paid to the increased internal rotation of the tibia and, if confirmed, its implications for the effects of long-term usage of knee braces. Additionally, the underlying effects that may cause the observed kinematic changes should be clarified by analyzing the impact of different brace types on neuromuscular activation patters. Thereby, once again, insights on long-term effects of brace usage on muscular strength and subsequent muscular joint stabilization are needed.

Finally, it should be mentioned that improved knee stability could also be obtained through reconstruction of the ACL, but the incidence of osteoarthritis 11 years after operative management is much higher than after conservative treatment (Kessler et al., 2008). In consequence, conservative therapy of the knee including bracing might be a cheap and efficient alternative, to (a) reduce the loss of proprioception and mechanical stability, (b) maintain physical activity levels and (c) prevent secondary injuries like meniscus tears or osteoarthritis. The results of the present study, therefore, are of particular relevance, not just for scientists and therapists, but also for the health care system, brace companies, and the patients themselves.

## Data Availability Statement

The datasets generated for this study are available on request to the corresponding author.

## Ethics Statement

The studies involving human participants were reviewed and approved by Ethics Board of the State Medical Association of Baden-Württemberg. The patients/participants provided their written informed consent to participate in this study.

## Author Contributions

AF designed the study, collected and analyzed the data, evaluated the literature, and also wrote the initial draft of the manuscript. HS collected and analyzed the data and revised the manuscript. FM analyzed the data and revised the manuscript. SR evaluated the literature and revised the manuscript. SS revised the manuscript. WP was responsible for funding acquisition, designed the study, and revised the manuscript. TS was responsible for resources, supervision, and project administration, designed the study, and revised the manuscript. HS, FM, SR, SS, WP, and TS approved the final version of the manuscript. All authors contributed to the article and approved the submitted version.

## Conflict of Interest

The authors declare that the research was conducted in the absence of any commercial or financial relationships that could be construed as a potential conflict of interest.
